# A Vision for the Future of Multiscale Modeling

**DOI:** 10.1021/acsphyschemau.3c00080

**Published:** 2024-03-04

**Authors:** Matteo Capone, Marco Romanelli, Davide Castaldo, Giovanni Parolin, Alessandro Bello, Gabriel Gil, Mirko Vanzan

**Affiliations:** † Department of Physical and Chemical Sciences, University of L’Aquila, L’Aquila 67010, Italy; ‡ Department of Chemical Sciences, University of Padova, Padova 35131, Italy; § Department of Physics, Informatics and Mathematics, University of Modena and Reggio Emilia, Modena 41125, Italy; ∥ Instituto de Cibernética, Matemática y Física (ICIMAF), La Habana 10400, Cuba; ⊥ Department of Physics, University of Milano, Milano 20133, Italy

**Keywords:** multiscale modeling, multiscale simulation, QM/QM, QM/MM, QM/Continuum, photosynthesis, computational chemistry

## Abstract

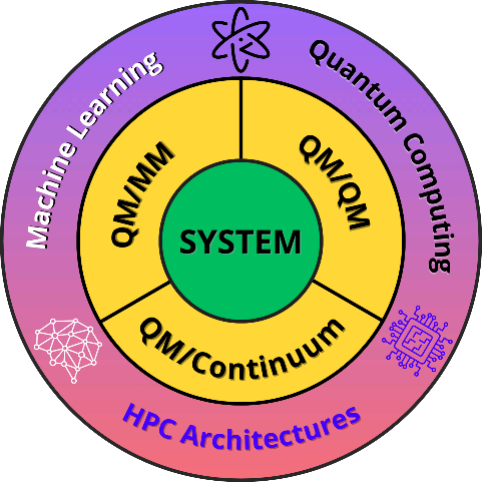

The rise of modern computer science enabled physical chemistry to make enormous progresses in understanding and harnessing natural and artificial phenomena. Nevertheless, despite the advances achieved over past decades, computational resources are still insufficient to thoroughly simulate extended systems from first principles. Indeed, countless biological, catalytic and photophysical processes require ab initio treatments to be properly described, but the breadth of length and time scales involved makes it practically unfeasible. A way to address these issues is to couple theories and algorithms working at different scales by dividing the system into domains treated at different levels of approximation, ranging from quantum mechanics to classical molecular dynamics, even including continuum electrodynamics. This approach is known as multiscale modeling and its use over the past 60 years has led to remarkable results. Considering the rapid advances in theory, algorithm design, and computing power, we believe multiscale modeling will massively grow into a dominant research methodology in the forthcoming years. Hereby we describe the main approaches developed within its realm, highlighting their achievements and current drawbacks, eventually proposing a plausible direction for future developments considering also the emergence of new computational techniques such as machine learning and quantum computing. We then discuss how advanced multiscale modeling methods could be exploited to address critical scientific challenges, focusing on the simulation of complex light-harvesting processes, such as natural photosynthesis. While doing so, we suggest a cutting-edge computational paradigm consisting in performing simultaneous multiscale calculations on a system allowing the various domains, treated with appropriate accuracy, to move and extend while they properly interact with each other. Although this vision is very ambitious, we believe the quick development of computer science will lead to both massive improvements and widespread use of these techniques, resulting in enormous progresses in physical chemistry and, eventually, in our society.

## 1 Introduction

**Figure 1:**
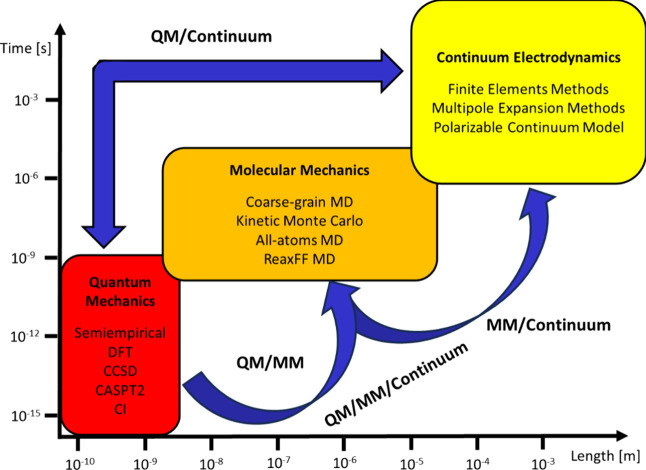
Application domains of the various computational methods typically used in physical chemistry. Cartesian axes refer to the space-time scale involved in the process under investigation. A list containing some of the most frequently used techniques for a certain scale domain is provided within the boxes. Blue arrows depict the main multiscale approaches commonly adopted in the study of physical-chemical phenomena. Acronyms MD, DFT, CCSD, CASPT2, and CI stand for Molecular Dynamics, Density Functional Theory, Coupled-Cluster up to Single and Double excitations, Complete Active Space second order Perturbation Theory, and Configuration Interaction, respectively.

The past century was characterized by enormous scientific revolutions. Formulation of theories such as quantum mechanics and general relativity dramatically changed the way we approach natural phenomena and have profoundly modified how we understand science.^1,2^ Hence, it is somewhat ironic to think that, by the end of the XIX century, a significant part of the scientific community considered the search of the fundamental laws of nature almost complete.^3,4^ One of the reasons behind this confidence was assuming that the universe could be described through the very same mathematical equations at any different space and time scale. In chemical sciences, this belief collapsed as the scientific progress of the XX century unravelled how, at the atomistic scale, most of the experimental observations deviate from what is expected by classical physics, leading in turn to the emergence of a whole new theoretical framework to be effectively rationalized in terms of quantum mechanics (QM).^5^ Such a new set of laws and concepts allowed natural processes at the molecular level to be described with unprecedented precision and accuracy, but were unsuitable for dealing with most of the phenomena occurring at macroscopic scale. This drawback quickly prompted the scientific community to explore different space-time scales with appeal to different sorts of approximation and this is the reason why in contemporary physical chemistry, and in particular when simulating complex molecular systems, we use a variety of theoretical approaches to study physical phenomena. As illustrated in Figure 1, depending on the length and time scales involved we can use first principle methods based on QM when inspecting fundamental features of matter, classical Molecular Mechanics (MM) when looking at the dynamics of complex molecular and supramolecular structures, and classical electromagnetism to model the macroscopic optical response of extended surfaces, bulk materials, or nanoparticles.^6^

The rapid development of computer science over the past decades allowed these approaches to be applied in the study of progressively larger and more complex systems, giving rise to the field of computational chemistry.^7^ This discipline has become increasingly important as it allows us to directly look at phenomena occurring at the molecular scale, providing new information on systems’ properties and dynamics, which in turn promoted massive advances in chemical sciences. Unfortunately, despite the computational power and the algorithms efficiency have steadily increased along the years, for several phenomena an accurate modeling is still unfeasible. Many processes occurring at the molecular or supramolecular level such as catalysis, electron transport, or optical excitations require a full quantum treatment to be properly studied, as they involve particles (like electrons and photons) and/or quasi-particles (such as phonons, plasmons, excitons) which feature an intrinsic quantum nature. In the meantime, macroscopic environments (solvents, substrates, nanoparticles, etc.) can have a great impact on the electronic and optical properties of molecules, for instance being responsible for stabilizing ground states, shifting excitation energies, or tuning excited states optical activity.^8,9^ Accounting for the effect of the environment is a major challenge in computational chemistry, as the breath of spatial and temporal scales involved in the processes do not permit first principle calculations of the whole system, due to the large collection of atoms and molecules needed to produce accurate results.^10−12^ For these reasons, since the dawn of computational chemistry there has been the urgency to devise clever means that, on one side, account for the physics of the environment and, on the other, can explore different spatiotemporal scales, representing a feasible alternative to the full ab initio scheme. A very successful kind of such “clever means” is represented by multiscale modeling, which consists in combining different methods to treat different segments of the system.^13^

The foundation of this technique dates back to the 1970s, when a series of works by A. Warshel, M. Levitt, and M. Karplus showed that hybrid methods, combining classical and quantum techniques, could be effectively adopted to describe the property of molecular systems. A first remarkable accomplishment was obtained in 1972 when Warshel and Karplus predicted the optical and vibrational spectra of π-conjugated molecules by modeling the π-electrons through a semiempirical approach,^14^ while including the presence of σ-electrons and nuclei using a classical framework.^15^ After a few years, Warshel and Levitt studied the stabilization of a carbocation within the active site of lysozyme using a partitioning scheme where, depending on the part of the system under study, electrons could be treated either as classical or quantum particles, performing de facto the first ever QM/MM calculation.^16^ In the meantime, they also proposed the idea of coarse-graining an MM simulation of a biological structure by grouping some specific protein atoms into beads, each described as a single classical unit, leading to the first original simulation of a protein folding.^17^ Those seminal works showed that by combining different approximations and effective theories it is possible to increase the size of the system under study without sensibly compromising the accuracy of the calculations, paving the way for modern multiscale modeling. Because of that Warshel, Levitt, and Karplus were awarded the Nobel prize in chemistry in 2013.^18^

Thanks to the enormous advances in high-performance computing and to the huge developments of computational algorithms reached in the past decades, multiscale modeling has become the workhorse of physical chemistry, constantly providing insights on countless chemical problems of much scientific interest.^19−21^ Furthermore, as it continues to evolve and adapt, it crosses the borders of chemistry and becomes an interdisciplinary technique, commonly adopted in fields like biochemistry,^22,23^ computer science,^24^ nanoscience,^11,25^ drug discovery,^26^ and materials science.^27,28^ The capability to contribute in such diverse yet technologically relevant fields makes multiscale modeling one of the most powerful tool we have to address some of the major challenges of our time, such as global warming, energy supply, and disease control.^29,30^

In this vision, we first systematically review the main multiscale modeling approaches employed in physical chemistry to date, namely QM/Continuum, QM/MM, and QM/QM. We quickly go through the description of QM/MM/Continuum methods, which combine different aspects of the more traditional mentioned methods, while intentionally omitting any specific reference to MM/Continumm approaches since they are less employed within physical chemistry and akin fields of research, even though there are notable exceptions (see e.g., ref (31)). We then conclude this section with an overview of the state of the art of new techniques such as Machine Learning (ML) and Quantum Computing (QC), showing their potentialities and highlighting how they are already impacting the field.^32,33^ The following part of this work is devoted to illustrate our vision about the evolution of multiscale modeling in the next decades. Here, we propose a novel calculation approach based on the use of multiple mobile QM centers, which can move and extend accordingly to the phenomenon under investigation, while properly interacting with the other simulation domains. As a test case example, we analyze how this method could provide valuable information on natural photosynthesis and thus boost the research on light-harvesting devices. After focusing on the evolution of the discipline itself, in the last part of this work we give our opinion on how multiscale modeling may become a widespread tool in academia and industry, by the emergence of increasingly user-friendly software and its dissemination among young students and researchers. We believe that such advancements will make multiscale modeling an indispensable tool when dealing with the rationalization of physicochemical phenomena.

## 2 State of the Art

An accurate modeling of chemical phenomena such as reaction mechanisms, charge and energy transfers, or optical excitations, requires a high level description of the molecular species involved. That is because those processes strongly depend on the electronic structure of the system, which must therefore be treated at a state-of-the-art level of accuracy, while considering the environment under certain approximations. Thus, the most relevant molecular species require a full QM treatment, either within the framework of Density Functional Theory (DFT) and time-dependent DFT (TDDFT) or through wave function based methods, such as Configuration Interaction (CI), Coupled Cluster (CC), and so on. However, the description of the environment could rely on many approaches, but the main ones adopted in computational physical chemistry are dielectric continuum models, MM, and effective QM treatments, which we will cover in detail in the following. In all mentioned methods, independent simulations performed at different levels are effectively coupled, and this classifies them as parallel (or concurrent) modeling techniques. In such approaches it is possible to perform interconnected calculations by treating different regions of the systems concurrently with different levels of theory, and thus capturing the interplay between distinct scales in real time. Such methods include interactions between different domains, enabling the emergence of collective behaviors and potentially tackling the dynamic evolution of the entire system.^34^ Alternatively, sequential modeling is characterized by a stepwise progression through different levels of theory. Here the phenomenon is studied with approximate (though computationally feasible) methods, such as classical MM force fields or semiempirical QM approaches, the latter including information coming from more sophisticated simulations. This is, for example, the case of Molecular Dynamics (MD) simulations performed with empirical potentials whose parameters were previously fitted on data coming from QM calculations. Such an approach allows a reasonable compromise between computational efficiency and accuracy, and can be selectively tailored to specific regions or features of the system. However, a limited number of parameters cannot incorporate the whole physics of a molecular system, and thus these methods are more subject to methodological errors, which can also accumulate during the interdomain information transfer.^19^

We have intentionally chosen to omit a detailed discussion about sequential modeling, focusing instead on the main branches of parallel modeling, namely QM/continuum, QM/MM, and QM/QM, which in our opinion represent the most promising techniques within the world of multiscale modeling in physical chemistry.

### 2.1 QM/Continuum

**Figure 2:**
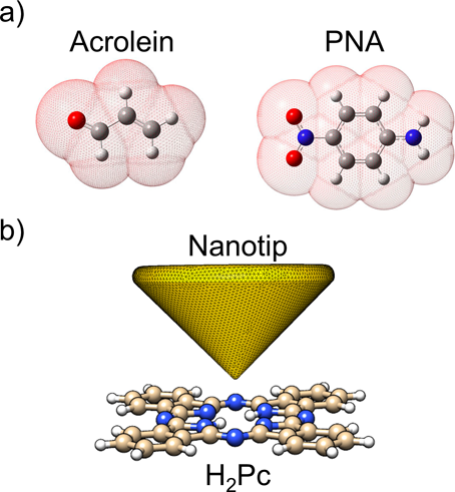
(a) Two different QM chromophores surrounded by the corresponding PCM cavities to model the solvent response: Acrolein and *para*-nitroaniline (PNA). Adapted with permission from ref (41), Copyright 2020 AIP Publishing. (b) Illustration of the PCM-NP^36,42^ theory applied to model a plasmonic gold tip interacting with a phthalocyanine molecule (H_2_Pc). The surface of the tip is discretized to numerically solve the PCM problem.

In QM/continuum models we have to consider physical interfaces separating the focus QM region from the environment, with the latter to be treated within classical electrodynamics. Such interfaces demarcate spatial regions associated with different continuous media, i.e., endowed with different frequency-dependent dielectric functions, with the focus QM region typically tackled as if it were in vacuum. Surely, one of the first and most successful dielectric continuum approaches is the so-called Polarizable Continuum Model (PCM) as applied to solvated molecules, where a realistic cavity (conforming with molecular shape) is built around a solute molecule, separating it from a homogeneous solvent^35^ (Figure 2a). The scope of the PCM framework however is wide and nowadays covers many other kinds of multiscale systems beside solvated molecules, such as molecules in proximity to metal nanoparticles^36,37^ or scanning electron microscopy tips (Figure 2b),^38^ nanoclusters embedded in a solid matrix,^39^ and molecules adsorbed on substrates,^40^ just to name a few.

#### 2.1.1 PCM: The Basic Model

PCM allows to find an approximation for the electrostatic (or quasi-electrostatic) interaction between the environment and the target molecule, via the solution of a generalized Poisson problem for the potential ϕ(**r**) produced by the molecular charge density ρ(**r**) in a segmented domain. In the simplest case of a single interface enclosing the source region Ω (e.g., a vacuum cavity embedded in a solvent), we need to solve
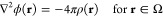
1
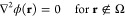
2imposing the continuity of the electrostatic potential and the normal component of the electric displacement vector across the interface Γ, separating Ω from its complement.^35^ Such a solution can be written as a sum of the molecular potential in vacuo, *v*(**r**), plus a polarization contribution *w*(**r**), i.e.,
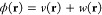
3

In turn, the polarization potential may be written as,
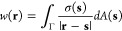
4where σ(**s**) is an Apparent Surface Charge (ASC) density supported at the interface Γ. Numerically, PCM is a Boundary Element Method (BEM), whereby the latter ASC density is discretized in a collection of (*T*) point charges (*q*_*i*_) sitting at the nodes (**s**_*i*_) of a proper tessellation of Γ. The BEM version of eq 4 hence reads
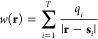
5

In general, polarization charges {*q*_*i*_} are obtained from a linear response equation, given the molecular field or potential. There are several flavors of PCM, depending on the degree of approximation of the polarization response that is used and of the quantity we want the system to respond to (electrostatic potential or field). One of the most successful versions of PCM is the Integral Equation Formalism (IEF) PCM,^43^ according to which polarization charges are calculated as
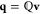
6where  is a response matrix depending on the *static* dielectric constant at each side of the interface and its geometry, whereas **q** and **v** are arrays of polarization charges and the molecular potential values on each tessera, respectively.

Another dielectric continuum approach, popular in the community studying solvation effects, is the COnductor-like Screening MOdel (or COSMO). Such a model starts by approximating the continuum as a conductor (i.e., with ϵ = *∞*) and then rescales the obtained unscreened polarization charges by an empirical function *f*(ϵ) which depends on the actual dielectric constant of material (ϵ < *∞*).^44^ In practice, the COSMO alternative to eq 6 is
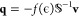
7where  stores the BEM representation of the Calderon operator.^35^

In cases with a single interface, such as those of solvated molecules or molecules physisorbed on metallic nanoparticles, the only source of the applied potential is the molecule, whereas in cases with more than one interface (e.g., ref (45)), there is a further contribution coming from polarization charges at other interfaces.

Within PCM, the target molecule induces a polarization in the environment which, in turn, polarizes the molecule. Therefore, to couple PCM to quantum chemistry methods, the Hamiltonian for the target molecule should be modified as follows:^46^
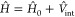
8where  is the Hamiltonian of the isolated molecule and  reflects the interaction with the environment, which depends on the state of the molecule itself. In general, such an additional term covers the interaction with electrons and nuclei, yielding
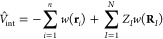
9where *n* and *N* are the number of electrons and nuclei of the molecule, respectively, and *Z*_*I*_ the atomic charge of the *I*-th nucleus. The free energy contribution coming from the environment interaction can thus be written as
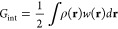
10which is interpreted as the work needed to assemble the molecular distribution of charge ρ(**r**) in the presence of the environment.

Under the Born–Oppenheimer approximation, the electronic Hamiltonian incorporates only the first term in eq 9, which involves a polarization potential due to both electrons and nuclei, acting on the electrons from the target molecule. The time-independent electronic problem may be written as the nonlinear Schrödinger eq, i.e.,
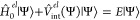
11where |Ψ⟩ and E are the stationary electronic state and energy, respectively, of the target molecule embedded in the environment, and  and  are counterparts of  and  acting on electrons. We stress the nonlinearity of eq 11 by placing the electronic state as an argument of the interaction Hamiltonian, i.e., . Indeed, to obtain the electronic ground state of molecules embedded in the environment, we need then to solve a self-consistent problem, whereby the electronic ground state at iteration *n* ≥ 1, |Ψ^(*n*)^⟩, is an eigenstate of the Hamiltonian , given . The ground state problem is variational in nature, as it can be formally obtained from the minimization of the proper free energy functional.^35,46^

#### 2.1.2 PCM: Some Extensions

PCM is a mature theoretical framework for considering (quasi-)electrostatic interactions of molecules embedded in polarizable environments.^47^ Over the last four decades or so, such an approach has been updated and refined to attain faster and more accurate results, as well as extended to cover a wider range of phenomena within chemical physics.

In particular, the basic model we just presented is useful when considering the stabilization of molecular ground states in solution, but it is insufficient to explain other effects, such as solvatochromic shifts.^48^ To that end, a nonequilibrium theory of the excited states of molecules embedded in a dielectric environment was formulated, whereby the polarization of the environment is only partially equilibrated with changes in the molecular state due to electronic transitions.^48,49^ Such a theory considers the inherent frequency dependence of the environment dielectric function, however approximately, by using two different (and real) dielectric constants, one active at zero frequency (static dielectric constant) and another at optical frequencies (dynamic dielectric constant). Following a further approximation much akin to the Franck–Condon principle, the theory assumes that only the faster (electronic) degrees of freedom of the environment polarization are able to respond to electronic excitations of the molecule, whereas other slower (nuclear) degrees of freedom lag behind, and remain equilibrated with the molecular ground state, just after the sudden transition.^35^ The faster polarization response is computed from the dynamic dielectric constant, whereas the slower counterpart depends on both, the static and dynamic dielectric constants.^49^ This scheme has been coupled to CI calculations, where for each excited state of the target molecule in vacuum, one solves iteratively eq 11, given a partition of the molecule–environment interaction potential into a slower component, frozen and independent of the excited (but not on the ground) state, and a faster contribution, which is state specific and changes during the iterative procedure.^48^

This approach to nonequilibrium effects of environment polarization on molecular excited states was first set up to tackle solvated molecules, as it is often the case, but it has also been adopted in more complex systems, such as solvated molecules physisorbed on nanoparticles,^50^ and on substrates,^40^ in these occasions coupled with linear-response and real-time TDDFT, respectively. However, depending on the specific application, such a paradigm may be inadequate to couple molecules with dielectric environments that are optically active in the relevant frequency range.

To overcome these drawbacks, one must take into account the polarization response of the environment in terms of its complex, frequency-dependent dielectric functions.^36^ The latter has been approximately achieved in a time-dependent treatment of nonequilibrium polarization by using a Debye dielectric model for solvents,^51^ and a Drude-Lorentz dielectric model for metal nanoparticles.^52^ The strategy behind such a treatment allows one to simulate the evolution of polarization charges coupled to the molecular potential, by propagating them according to a model-dependent equation of motion (EOM). Realistic (and generic) frequency-dependent profiles of complex dielectric functions are also recently accessible to PCM calculations in the EOM formalism, provided that a Drude-Lorentz fit of a dielectric function dependent quantity is available.^53^ This approach has been interfaced with real-time electronic wave function dynamics of the target molecule, where the time-dependent state is expanded in a suitable basis of CIS (CI up to single configurations) excited states, subject only to a frozen polarization of the environment in equilibrium with the ground state.^54^

There are many other unrelated developments within the PCM framework that can also be exploited by multiscale systems, such as considering interfaces defined by molecular charge isodensities,^55^ nonelectrostatic dispersion and repulsion interactions,^56^ anisotropic dielectrics described by a tensorial dielectric constant and ionic solutions described by a linearized Poisson–Boltzmann problem,^43^ diffusive interfaces between media modeled through a smooth and position-dependent dielectric function across the boundary,^57^ nonlocal electrostatics effects,^58^ etc. We have mentioned the coupling of PCM to CI and DFT approaches, but there are several other quantum chemistry methodologies that have been successfully linked to PCM, such as Hartree–Fock (HF), Møller–Plesset (MP), Coupled Clusters (CC), multiconfigurational and multireference methods, and so on. To date, many of the codes commonly used in computational chemistry (Gaussian,^59^ GAMESS-US,^60^ Octopus,^61^ etc.) contain some feature or other of PCM.

#### 2.1.3 Beyond PCM: Retardation Effects

Earlier, we emphasized that PCM is a quasistatic method. Indeed, it proceeds from the solution of Poisson equation for the scalar (electrostatic) potential. This formalism is approximately valid only when the oscillation period of the time-dependent electric fields involved is larger than the time it takes for light to cover a characteristic distance within the system, *ωd*/*c* < 1. Otherwise, one needs to solve the full set of Maxwell equations. Using Lorentz gauge, a simplified system of inhomogeneous Helmholtz equations for the scalar and vector potentials, ϕ(**r**) and **A**(**r**), stems out of Maxwell equations in frequency domain, i.e.,^62^
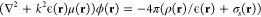
12
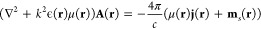
13where *k* = ω/*c*, ρ(**r**) and **j**(**r**) are densities of charge and current which are the sources of the potentials, whereas σ_*s*_(**r**) and **m**_*s*_(**r**) are surface densities of charge and current arising from discontinuities in the dielectric permittivity, ϵ, and/or magnetic permeability, μ, across space (namely, due to physical interfaces), respectively.. In the case of a single interface, the latter pair of equations can be solved by means of the following ansatz, much akin to eq 4,
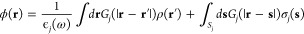
14
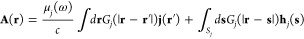
15where *S*_*j*_ is the boundary between media *j* = 1, 2, ϵ_*j*_ and μ_*j*_ are the medium-*j* dielectric permittivity and magnetic, respermeabilitypectively, , and *G*_*j*_(|**r** – **r**′|) is the Green function of the Helmholtz operator, (∇^2^+*k*_*j*_^2^). σ_*j*_ and **h**_*j*_ are boundary surface densities of charge and current which takes into account the jump conditions for the potentials and their derivatives at the physical interfaces. Unlike σ_*s*_ and **m**_*s*_, σ_*j*_ and **h**_*j*_ assume a different value at each side of the interface.^63^

Starting from eqs 14 and 15, a new nonquasistatic (or retarded) BEM formulation has been laid down, which finds the proper σ_*j*_ and **h**_*j*_ from which the modified potentials can be computed.^62,63^ A standard code implementing this method is the Matlab toolbox MNPBEM.^64^ Such a retarded BEM approach has been coupled to single-molecule emitters, described as few-level systems.^65,66^ Although, to the best of our knowledge, there are no current strategies to couple retarded BEM with an atomistic QM description of molecules, it is part of our vision that in the near future such an approach will be available to study long-ranged chemical physics phenomena within multiscale systems.

### 2.2 QM/MM

**Figure 3:**
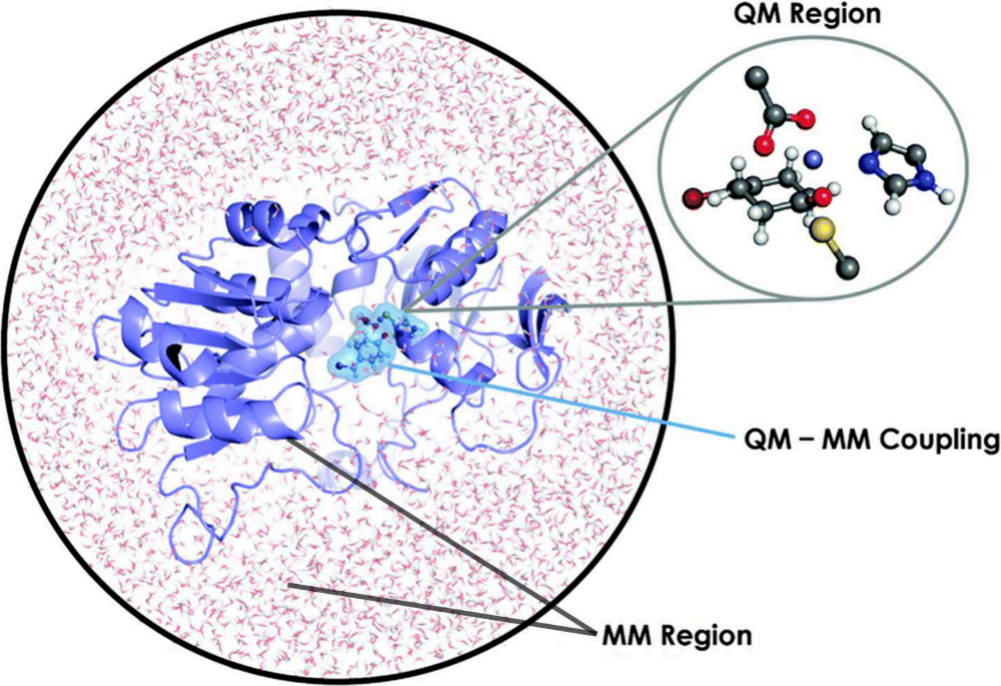
Pictorial sketch of a QM/MM partitioning scheme for a biocatalyic active site surrounded by water molecules. Adapted with permission from ref (76), Copyright 2016 Royal Society of Chemistry.

When modeling electronic processes in complex environments, it is often necessary to include an atomistic description of the region surroundings the site where the main event takes place. Indeed, highly anisotropic intermolecular interactions (like H-bonds or dipole–dipole coupling) between the focus system and its environment are known to have non-negligible effects on the local system dynamics,^67^ and require a proper treatment. In this regard, hybrid QM/MM methods are often desirable.^68−75^ The strength of such approaches relies on performing QM calculations on the part of the system where relevant electronic processes such as charge transfers or bond breakage/formation take place, while treating the remainder at the classical MM level (see Figure 3). In doing so, a fair compromise between accuracy, computational cost, and system size can be reasonably achieved, thus making it possible to model systems of realistic complexity.

Despite their well-established potential and widespread use, QM/MM methods have relevant drawbacks such as nontrivial implementation, poor computational scaling (in particular for the QM part), and complications in handling the boundary region (see Sec. 2.2.3). Through the years, several schemes have been proposed to evaluate the system total energy, which essentially needs to account for the energy of the QM region, the energy of the MM region, and their reciprocal interaction. The first two contributions are straightforward to address through the respective QM and MM calculations, while the interaction energy term is more subtle and deserves particular attention. Over the years different approaches have been proposed to calculate this quantity, and they can be approximately divided into two categories, namely subtractive and additive schemes, each of which can be described at different levels of accuracy and sophistication.

The various QM/MM schemes discussed below can be found today in widely used quantum chemistry codes, such as Gaussian,^59^ GAMESS-US,^60^ NWChem,^77^ QChem,^78^ and ORCA.^79^

#### 2.2.1 Subtractive and Additive Schemes

In the subtractive scheme, the QM/MM total energy *E*_QM/MM_^sub^ is evaluated as
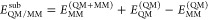
16where the superscripts between round brackets on the right-hand side of eq 16 refer to which region is described with the specified level of theory, the latter appearing as subscript. eq 16 clearly shows that in the subtractive scheme the coupling between the QM and MM regions is implicitly evaluated, and depending on the type of embedding approach adopted (see Sec. 2.2.2) different polarization effects between the two regions can be considered. The main idea behind eq 16, is to avoid double counting the contribution from the QM portion of the system. Generally, these schemes are easier to implement, especially in the case of mechanical embedding where QM and MM calculations do not have to communicate among each other, but only require the presence of a force field for the QM region. Moreover subtractive schemes easily allow additional refinements of the QM region by further subpartitioning. Different QM subregions can thus be easily treated with different QM theories, resulting in rather versatile matryoshka approaches. On this aspect, one of the most known and used QM/MM subtractive schemes is the ONIOM^68,70,80^ method, where both QM/MM and QM/QM boundaries are possible (see also Sec. 2.4).

On the other hand, when additive schemes are used the QM/MM total energy reads
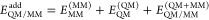
17where the interaction term between the two regions *E*_QM/MM_^(QM + MM)^ is explicitly evaluated at the QM/MM level. The way this contribution is evaluated strongly depends on the embedding scheme adopted. This brings further methodological complications with respect to the subtractive approach, especially when direct bonds between the boundary regions are severed (see Sec. 2.4).

#### 2.2.2 Embedding Approaches

As a first approximation, the interactions between QM and MM regions can be treated at the classical level i.e., using MM force fields. This embedding scheme, usually referred to as mechanical embedding, consists in modeling chemical bonds between QM and MM regions with classical harmonic potentials, whereas intermolecular interactions are evaluated using standard Lennard-Jones potentials and bare Coulombic electrostatic interactions. The latter are usually formulated in terms of atomic point charges that can be either defined according to standard MM force fields, or derived from ab initio calculations, e.g., via the so-called RESP charges.^81^ In this simplest scenario, the QM calculation is performed as if the QM subsystem was isolated in vacuum, and thus its electron density is not subjected to environmental perturbations. This picture is often unrealistic and oversimplified, especially when QM regions are in close proximity to highly polarized and/or charged parts of the MM scaffold. Given these reasons, a better description of the QM part can be attained using electrostatic embedding. Here, the MM point charges can directly polarize the QM electron density, because the electrostatic interaction between the two subsystems directly enters into the QM Hamiltonian. Even if this latter approach clearly constitutes an improvement over the mechanical embedding, the influence of MM atoms on the QM regions is usually limited to electrostatics and, most importantly, the MM region is not subjected to any influence from the QM electron density. Polarizable embedding schemes are currently being developed to fill this gap. In such a case not only the MM region can polarize the QM subsystem, but also the QM electron density can back-polarize the MM atoms, allowing a proper treatment of mutual polarization. Various approaches have been explored in this direction: some of them rely on introducing polarization effects through ab initio force fields, such as the Effective Fragment Potential^82^ (EFP) method, while others focus on developing classical MM polarizable force fields. Among the latter, the better known are based on Drude oscillators,^83,84^ fluctuating charges,^85−87^ or induced dipoles,^8,88−91^ which add some flexibility to the MM point charges, either dressing them with atomic polarizabilities or letting the MM charges fluctuate due to interactions with the environment.

Despite their more realistic description of QM/MM interactions, these models are neither easy to implement nor computationally cheap. Indeed, the inclusion of a mutual polarization implies that at each step of the QM self-consistent calculation, the polarization interaction has to be self-consistently evaluated, thus increasing the computational demand compared to simpler embedding schemes. Besides, the difficulty in developing accurate polarizable force fields as well as implementing the corresponding changes to QM codes has posed severe obstacle to their widespread use so far.^92^ Nevertheless, linear scaling approaches aimed at solving the polarization equations more efficiently are currently being developed, thus laying the groundwork for a much more wider use of polarizable embedding schemes in the near future.^93^

#### 2.2.3 Defining and Handling the Boundary Region

In general, partitioning the system is not a trivial task. First of all, the presence of a boundary between the QM and MM regions assumes the compatibility of the corresponding level of theories, and in particular on the reliability of the MM force field parameters, since they are usually optimized against different reference data.^69^ Moreover, while in many standard implementations of QM/MM schemes a static definition of each region is adopted, there are cases in which a dynamical definition of the boundary is preferable. Typical examples are MD simulations where the undergoing process causes some groups to move across the previously defined borders, such as for solvent molecules moving across the solvation shell of a catalyst, or for the sites involved in the transfer of atomic species.^94^ In these situations, a unique definition of the QM region results inappropriate, and the choice of the QM and the MM subsystems has to be made more flexibly, likely on-the-fly, in order to adapt to the actual atomic configurations. In this sense, adaptive QM/MM approaches have been recently devised and applied to analogous problems.^95^ However, this approach presents some significant challenges in handling transitions between potential energy surfaces, which are linked to different assignments of atoms and molecules to the QM and MM. The objective is to ensure a smooth atom exchange between these regions, maintaining consistency in energy, forces, and minimizing border effects.^95,96^ When partitioning a system, the easiest situation to handle is when no chemical bond is crossing the boundary, as in the case of a QM chromophore surrounded by a MM solvent. However, if chemical bonds are connecting species located in different regions, they need to be carefully separated upon partitioning. In this scenario, a common strategy is the so-called link-atom approach, where the dangling bond of the QM subsystem is saturated by an additional hydrogen (or in general monovalent) atom.^26^ The QM calculation is then performed on the saturated system, while the surrounding MM region does not perceive the presence of this fictitious atom. Despite being an efficient way of treating the boundary region, the link-atom approach also brings some technical complications. For instance, the position of the additional atom needs to be frozen to prevent nonphysical atomic movements during the simulation. Furthermore, in the case of electrostatic and polarizable embedding, the presence of the link atom can lead to an unrealistic polarization of the QM wave function due to the presence of point charges on the MM atom that is actually bonded to the QM region in the real system.

Other approaches, albeit less frequently used, are possible. Among them, it is worth mentioning the pseudobond approach,^97^ the localized hybrid orbital method^98^ (LSCF), and the generalized hybrid orbital^99^ (GHO) approach. The first relies on capping the QM dangling bond with a pseudoatom, whose properties are parametrized to reproduce most faithfully the features of the real system’s bond. The other two methods instead replace the missing bond with a doubly occupied localized orbital in the QM region (LCSF) or by including hybrid orbitals on the MM atom that is connected to the QM one (GHO). In all these cases, system-specific parametrization of pseudoatoms or local orbitals is required, which practically hinders a widespread use of these strategies compared to the link-atom approach.

### 2.3 Layered Approaches: Integrating Multidomain Simulations

**Figure 4:**
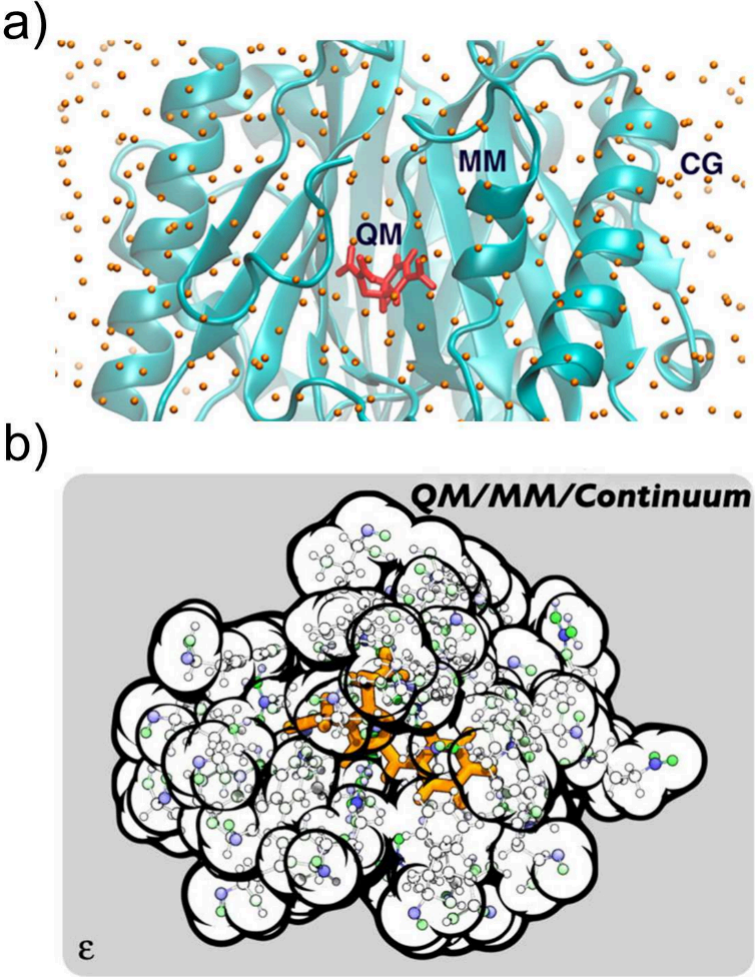
(a) Pictorial representation of a QM/MM/CG modeling setup. The chemically relevant active site is described at QM level, the surrounding biomolecular environment is treated using a MM force field whereas the solvent is modeled by a CG approach. Adapted from ref (105), Copyright 2016 American Chemical Society. (b) Schematic illustration of a QM/MM/Continuum description that could be used to model a QM active site embedded in the MM protein environment. The surrounding dielectric (e.g., solvent) is described by the continuum. Adapted with permission under a Creative Commons CC BY 4.0 License from ref (108), Copyright 2021 AIP Publishing.

QM/MM methods can quickly become computationally demanding not only when the QM region is sizable or when the QM level of theory is costly, but also when the MM region is so large and flexible that an extensive sampling of the environment degrees of freedom is required. In such cases, it may occur that multiple atomistic configurations of the environment could appear with similar probabilities in the thermodynamic ensemble, thus calling for multiple QM/MM calculations to obtain a faithful description. Hence, there have been recent efforts in combining QM or QM/MM calculations with a coarse-grained (CG) description of the environment,^100−107^ thus accounting for the degrees of freedom of the environment through an effective approach. A graphical example of such a multilevel approach is available in Figure 4a.

QM/Continuum models (discussed in Sec. 2.1) proved to be very efficient in evaluating statistically averaged environmental effects, without requiring an extensive sampling of the environment degrees of freedom. Nevertheless, they certainly lack the explicit description of short-range anisotropic interactions, which is something that can be correctly recovered by QM/MM methods. In this regard, QM/MM/Continuum methods have been recently developed (Figure 4b), trying to combine the most useful aspects of both QM/MM and QM/Continuum multiscale techniques while retaining a computationally feasible methodology, thus enabling an upscaling of systems that can be quantitatively investigated. Early attempts to combine discrete and continuum modeling of solvent molecules nearby a quantum center featured simple models of the continuum based on the dipolar approximation.^109−111^ However, it was soon realized that a more sophisticated model was necessary to achieve realistic results and thus PCM was later coupled with discrete QM/MM schemes.^112−116^ In the most recent QM/MM/PCM formulations^86,114,115,117−119^ the MM part is also polarizable, leading to a complex nonlinear problem to be solved as the induced polarization of the MM atoms are mutually coupled to the QM subsystem and to the PCM polarization charges, the latter usually being induced by both QM and MM atoms. Despite its complexity and its nontrivial implementation, QM/MM/Continuum calculations proved to converge faster than those obtained with a full QM/MM approach with respect to the size of the MM region and to MM cutoff radius length.^114^ Furthermore, they also were shown to improve the results of QM/Continuum approaches when dealing with QM subsystems strongly coupled with the environment,^115^ thus setting the stage for investigations of relevant QM regions that are subjected not only to strong local environmental anisotropic interactions, but also to long-range solvation effects.

### 2.4 QM/QM

**Figure 5:**
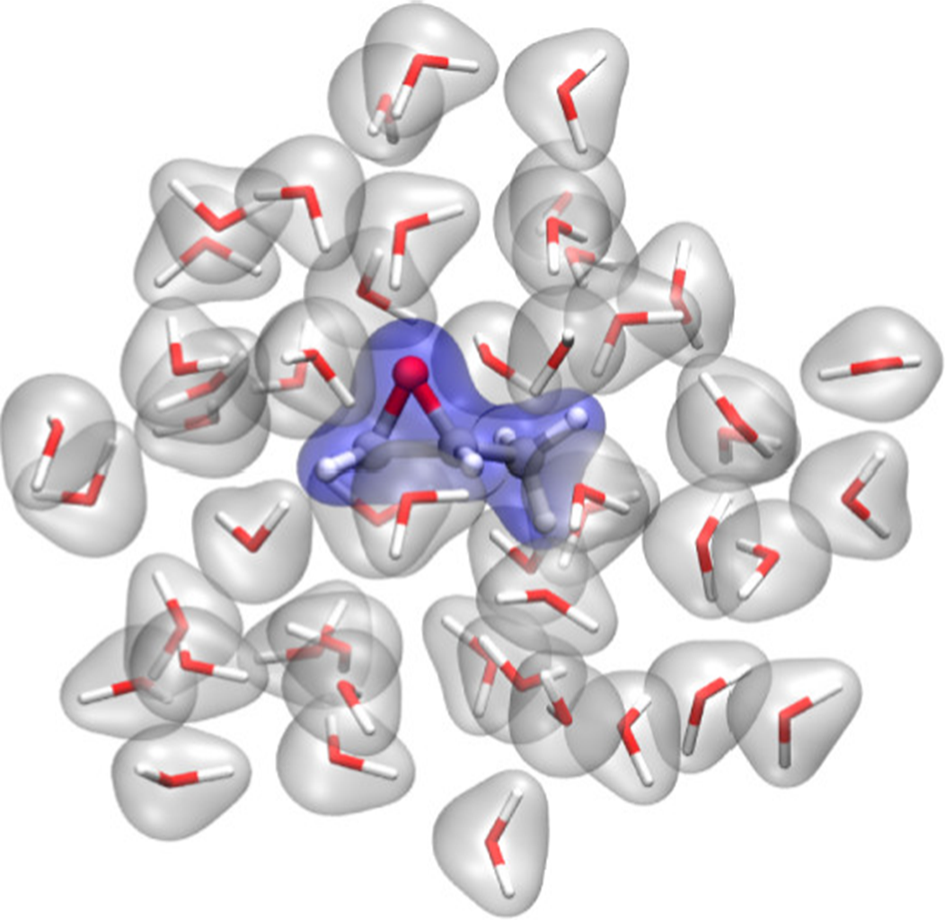
Pictorial representation of a QM/QM embedding scheme. Different degrees of accuracy at quantum mechanical level provide electronic densities with different precision (depicted with different colors). Adapted from ref (146), Copyright 2021 American Chemical Society.

As mentioned, some significant limitations hinder the widespread adoption and versatility of QM/MM methods, such as their lack of portability and difficult technical implementation.^20,26,120^ For instance, when the QM region does not feature standard force fields parametrization, ad hoc adjustments, either based on experimental data or quantum mechanical calculations, are necessary, thus resulting in a system-specific tailoring procedure. As a result, they may not be readily transferable to different systems without prior adaptation when applied to new scenarios. Furthermore, QM/MM methods are known to poorly describe nonelectrostatic interactions between the boundary regions, thus neglecting a proper account of quantum effects and correlation between the embedded subsystem and the environment, which often requires a proper description of Pauli repulsion and dispersion interactions, going beyond classical electrostatics.^121−123^ These quantum effects play a crucial role in certain chemical events of scientific interest, such as DNA/RNA stacking and resulting functionalities,^124^ solvatochromism,^123^ membrane processes, charge localization and transport,^125^ and agostic interactions in organometallic chemistry,^126^ just to name a few, making QM/MM investigations often inaccurate in these circumstances. To this end, quantum embedding methods have been designed to map the focus system into the quantum realm.^126−134^ In the same spirit as the QM/MM nomenclature, when we decide to treat our surroundings at the level of quantum mechanics, this multiscale strategy is referred to as QM/QM (see Figure 5).

Due to its outstanding compromise between computational cost and accuracy, DFT has long been used to model systems where an ab initio description of the phenomenon of interest is unavoidable, thus making it one of the mostly used quantum chemistry methods to date. Nevertheless, its common practical application is limited to systems composed of hundreds of atoms at most, and few cases have managed to go beyond that limit by resorting to Graphical Processing Units (GPUs).^135,136^ Clearly, this computational bottleneck limits the full applicability of standard DFT calculations to larger systems of biological relevance, thus calling for approximations to make such QM calculations practically feasible despite the system size.

Stemming from DFT, different partitioning and embedding schemes have been developed over the years, which all essentially rely on combining high-level DFT or other wave function based calculations (WF) on a small targeted portion of the system with a cheaper QM description of the surroundings, the latter often rooted in DFT-based approximations. Such schemes are commonly called DFT-in-DFT and WF-in-DFT QM/QM embedding methods, respectively. Partitioning of the system electronic degrees of freedom in all these methods is far from being trivial and different strategies have been adopted. However, the main criterion is defining active and inactive system regions where different levels of approximation are used and this division is often based on physical quantities of relevance, such as the density function, the density matrix or molecular orbitals. Among all DFT-in-DFT developments, we mention those that have attracted much scientific attention and active research over last decades, such as Subsystem DFT,^137^ Frozen Density Embedding Theory (FDET),^138,139^ Density Functional Embedding Theory (DFET)^140,141^ and Partition Density Functional Theory (PDFT).^142,143^ A common feature among these methods is that the embedding is actually taken into account by an effective embedding potential operator that enters into the Kohn–Sham equations of the target subsystem after partitioning. Energy minimization is then achieved by imposing further constraints on the total density, which is expressed as the sum of independent subsystems densities constituting the whole system. In this picture, each subsystem density can be computed from the corresponding Kohn–Sham orbitals, whose occupation number can either be an integer (as in FDET and Subsystem DFT) or a fractional number (as in PDFT), the latter being more suitable to describe chemical events where molecular dissociation takes place.^144^ The environment density can either be frozen throughout calculations, as it was in the initial form of FDET, or can be effectively optimized during optimization steps, as in the freeze-and-thawed FDET approach.^125^ Even though these approaches are suitable to recover quantum effects for systems where standard full DFT calculations are not feasible, they also bring further complications, such as the nonadditivity issue of the exchange-correlation and kinetic energy functionals,^125^ the latter being usually overcome by adopting partitioning schemes on the density matrix or by orbital projection.^125,145,146^

Despite its widely recognized success, DFT can also lead to unreliable descriptions, for instance when it is employed to model catalytic processes and reaction barriers involving transition metal complexes.^130,147−150^ In those cases, highly correlated wave function methods that focuses on a small system region containing the metal center, while treating the surroundings at DFT level, proved to be a viable and effective way to overcome DFT inaccuracies. These approaches, which follow as a natural evolution of DFT-in-DFT embedding schemes, describe environmental effects by using effective one-electron embedding potential that directly enters the wave function calculation through a modified effective one-electron Hamiltonian.^130,151−156^ Different wave functions methods within WF-in-DFT have been tested, such as Coupled-Cluster up to Single and Double excitations (CCSD), Complete Active Space second order Perturbation Theory (CASPT2), and Quantum Montecarlo (QMC). In the majority of those cases, WF-in-DFT calculations significantly improved over DFT-in-DFT descriptions.^151,153,157−161^ To conclude, we remark that several commercial and open-source codes contain implementations of the methods described so far (see e.g., Gaussian,^59^ ORCA,^79^ TURBOMOLE,^162^ MOLCAS,^163^ etc.).

### 2.5 Multiscale Simulations Aided by New Technologies

An increasing demand for computational capacity will be driven in the next decades by advancements in big data management and analysis, with massive consequences on the evolution of multiscale models.^42,164−166^ This urgent requirement is already inducing a shift in how we perceive computation. The limitation imposed by the on-chip power dissipation of current semiconductor technologies is hindering the development of traditional processing architectures, and we may witness a deceleration of Moore’s law. Consequently, diversifying computing paradigms, both in terms of architecture and algorithms, could prove advantageous for achieving a more sustainable balance between energy and material utilization. To some extent, this transition has already begun within the field of computational chemistry. In fact, active research initiatives are enhancing the performance and cost-effectiveness of simulating molecular Hamiltonians through the development of implementations on GPUs,^167−169^ and more recently, Tensor Processing Units (TPUs).^170^ Along the same line, quantum computation as applied to the simulation of quantum mechanical system represents a possibly hardware-accelerated option for future scientist in the field. To this extent, in this section we consider the main tools that have been developed so far to accelerate multiscale simulations with unusual hardware and/or modern software development techniques. For the sake of clarity, in the following, we distinguish between classical and quantum hardware/software advances.

#### 2.5.1 Machine Learning and modern High Performance Computing

The inclusion of GPUs and TPUs within high-performance computing ecosystems has deeply impacted the field of multiscale modeling. In particular, the last decades have witnessed the effort to provide specific support for multiscale packages toward GPU-based infrastractures.^171−175^ The next step we expect to observe in this context, not yet taken, is the transfer of these techniques onto TPUs. The latter are application specific integrated circuits and can be easily reprogrammed to efficiently perform linear algebra operations (by means of optimized libraries^176^). The impact these implementations could have in the future is still not clear, nonetheless recent works concerning quantum mechanical simulations^170,177,178^ show potential for unprecedented speedups in this context. Interestingly, these types of processors underlie the recent successes of ML, and the use of ML techniques is often accompanied by the application of standard multiscale methods with these architectures.^179^ Above all, one of the first results has been the development of learning pipelines (e.g., AlphaFold^180^) able to predict the folding processes of large proteins including ligands and cofactors.^181^ We imagine that the integration of these tools into multiscale simulations will be a key focus and will become widespread in physical chemistry in the coming decades. Indeed, the application of ML methods to multiscale modeling is a multifaceted process targeting the development and implementation of specific methodologies to be used, for instance, to optimize boundary regions between different partitions of the system,^182^ to obtain force fields for regions treated at the MM level,^183^ and to boost electronic structure calculations at the QM level.^184^ As mentioned, in this work we focus on parallel multiscale simulations, and thus we will not delve into the development of these latter methods or others such as Machine-Learned Interatomic Potentials (MLIPs).^185,186^ Nevertheless, given their importance we emphasize how MD simulations can benefit from MLIP, enabling simulations of systems composed of thousands of atoms, spanning propagation times up to 100 μs.^187^ This has the potential to open a new era for the simulations of long-lasting dynamical processes in increasingly larger and complex biochemical systems. With a partition like Figure 4 in mind, it is easy to realize that different ML methods may treat different regions of our system, ultimately posing a deep challenge in identifying key parameters for the problem under study. A potential solution is to combine deterministic and stochastic models and coupling the deterministic equations of classical physics, such as the balance of mass, momentum, and energy, with the stochastic equations typical of complex systems. Another option is to add reaction-diffusion equations that could help in guiding the design of computational models. Along those lines, physics-informed ML algorithms are promising approaches that inherently use constrained parameter spaces and constrained design spaces to manage very large systems in which physical quantities need to be conserved.^188^ The work summarized in ref (189) represents another promising approach. Here simulation parameters are learned and adjusted on-the-fly, building a data set of the simulated system at different scales. Particularly, continuum level calculations are used to machine learn CG potentials which in turn enable the production of a new data set, used to build an all-atoms simulation. We think that such a modular approach allows the flexibility needed to adjust over time (and space) the boundary region. This feature will be discussed in more detail in Sec. 3. However, to date, this type of methodology has not yet been tested to include a portion of the system treated at the QM level. In this perspective, we think the development of electronic structure methods based on ML, such as those using learning techniques to design exchange-correlation functionals,^190−192^ or Monte Carlo methods sampling the Hilbert space by through neural networks,^193−195^ will have a significant impact in this field. To conclude this section, we would like to emphasize how the evolution of multiscale techniques accompanying ML is likely to encounter QC methods. In the next section we will analyze the relation between QC and multiscale methods, but we will not consider another possible point of intersection between ML and QC: Quantum Machine Learning (QML).^196^ This research area aims to understand how to perform learning tasks using quantum computers on both classical or quantum-produced data. The variety of the methods that are being developed in this realm is remarkable and includes (i) engineering software and differentiation algorithms that can work on quantum hardware,^197−200^ (ii) bridging quantum information and learning theory,^201−204^ and (iii) broadening of the scope of possible applications.^205−207^ To date, it is difficult to predict how much this discipline will impact multiscale modeling, not only because sparse efforts have been spent in this direction (see for example ref (208)), but also because of the intrinsic issues emerging when those techniques are applied. The success of ML techniques indeed, depends on the empirical efficiency of the learning algorithms, whose theoretical scaling is difficult to analyze and, anyway, is not always promising.^209^ As it is not yet possible to perform sufficiently large learning tasks, the main developments within QML still remain theoretical, making any predictions about its practical impact far-fetched.^210^ Nevertheless, despite this premise, we believe that assessing the extent to which current ML algorithms can benefit from quantum acceleration is due and will have a sensible relevance in the future of multiscale modeling.

#### 2.5.2 Quantum Computing and Multiscale Modeling

**Figure 6:**
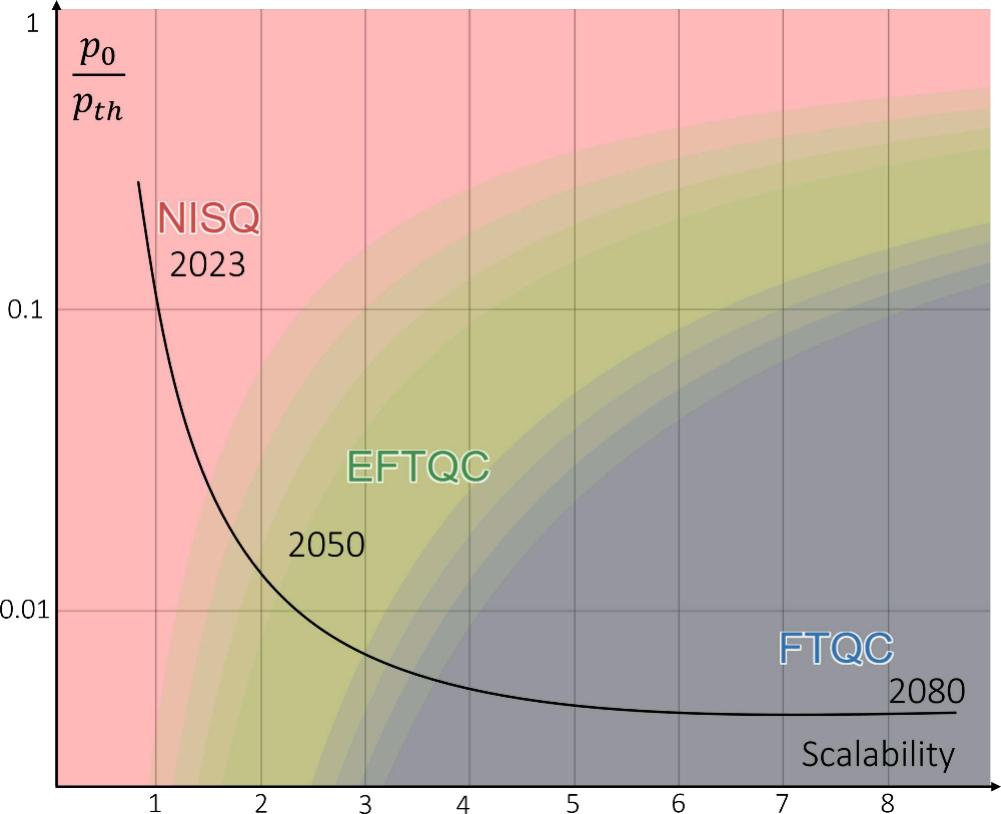
Possible quantum hardware progress according to a recently proposed scalability model.^215^ Outline of the regimes that QC are expected to go through in the coming years: (i) Noisy Intermediate Scale Quantum (NISQ, pink area), (ii) Early Fault-Tolerant Quantum Computers (EFTQCs, green area), and (iii) Fault-Tolerant Quantum Computers (FTQCs, gray area). Different stages of hardware development support different algorithms; in the former the main algorithms are variational, in the latter interference methods are more prominent. The intermediate stage consists in processors that modulate a circuit repetition/depth trade-off. Black solid line qualitatively poses a prospective hardware development timeline. Generated with ref (218) as per in ref (215).

The application of quantum computation to chemical systems is considered one of the possible paths to achieve quantum advantage on an industrial scale.^211,212^ Such a technology should dramatically improve the scalability of standard wave function methods, as we expect an exponential growth of the memory space of a quantum processor as its size increases. In analogy with standard computer science where the minimum unit of storage is in bits, in QC such minimum units are called qubits i.e., programmable two-level quantum systems which, due to their quantum nature, allow one to develop algorithms^213^ based on exquisitely quantum effects, not classically available. Naturally, the development of a specific hardware promotes the design of algorithms that best suit the available machine, in order to maximize the performance and this is also the case in the field of quantum computational chemistry.^214^ For this reason, in order to discuss the progresses and prospects of quantum multiscale modeling (i.e., multiscale modeling on quantum devices) we need to understand what kinds of quantum processor we expect to have in the coming decades. In Figure 6, we represent the possible development of quantum hardware according to ref (215). Discussing the assumptions and the possible quantitative accuracy of this evolution goes beyond the purpose of this work. Here we only use the results of this study to identify three different regimes of QC.^215^ As the quality of the individual qubits (expressed as the ratio , it quantifies the error rate during individual qubit operations, *p*_0_, against the correctable error rate using error correction protocols, *p*_*th*_) and the quality of the architectures increase (quantified with a scalability parameter), we can distinguish among: (i) Noisy Intermediate Scale Quantum^216^ (NISQ) devices, (ii) Early Fault-Tolerant Quantum Computers^215^ (EFTQC) and, (iii) full-fledged Fault-Tolerant Quantum Computers^217^ (FTQC).

In this section, we analyze the multiscale methods that have been developed in these areas and we discuss what tools a scientist in the coming decades will potentially be able to harness (or will have to develop) to leverage these technologies. Before we start, it is important to notice that, whether we are considering algorithms for current quantum computers or algorithms that can leverage error-correction, to simulate molecular systems with quantum computers requires devising a mapping to express the molecular wave function in terms of the quantum computer’s wave function. Even though not always considered, this represents per se an important step that can deeply affect the ultimate performance of the calculation. The vast majority of the algorithms developed during the last years focus on implementations and analysis using the Jordan-Wigner^219^ approach, due to its intuitive construction that maps the occupation of single-particle wave functions locally into the state of each single qubit and keeps track of the parity of the overall wave function in a nonlocal fashion. Nonetheless, our perspective on this matter is that current and future scientists should develop strategies to adapt the algorithm to the architecture, depending on the hardware/problem Hamiltonian pair at hand and on the best system-to-logical-to-physical mapping.^220^ Modular strategies as the ones developed in ref (221) are paving the way for development in this direction.

##### 2.5.2.1 Quantum Multiscale Modeling on NISQ

Given the characteristics of NISQs, the algorithms that can be implemented on these types of devices involve very shallow circuits where quantum coherence is not degraded by the external environment. In this context, the most commonly adopted algorithms are variational. In variational quantum algorithms^222^ one encodes a parametric trial wave function in a quantum computer and performs a classical optimization of the wave function parameters. When aimed to find the ground state of a quantum Hamiltonian, these algorithms are usually referred to as Variational Quantum Eigensolvers (VQEs).^223^ These algorithms usually seek to optimize a cost function *C*(θ) of the form:
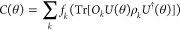
18

If we consider the solution of the electronic structure problem *k* = 1, *O*_*k*_ is the molecular Hamiltonian *H*_mol_, ρ_*k*_ is the processor initial state, and *U*(θ) is the parametrized unitary transformation applied to the initial state.

Given the resemblance of these algorithms to reinforcement learning and optimal control theory,^224,225^ various strategies have been borrowed from these domains to enhance both the performance and the range of applicability of these methods.^226,227^ Typically, these methods have an asymptotic runtime of  where *K* is a constant depending on the particular method (and system) considered and ϵ is the accuracy threshold we impose when solving the given task. Due to this asymptotic scaling, whether it is possible to demonstrate quantum advantage with these methodologies is still an open question.^228^ In the past few years, there have been considerable efforts to incorporate these approaches into the framework of multiscale modeling. Particularly, considering QM-continuum strategies, in ref (229) the VQE has been coupled with IEF-PCM^43^ to compute molecular properties in solution. Along the same line, an implicit description of the solvation effects via the Reference-Interaction Site Model^230^ has been integrated with the VQE.^231^ Moving to QM/MM methods, it is worth mentioning how NISQs devices have been leveraged using both subtractive^232^ and additive^233,234^ schemes to study biochemical and excited states problems. Multiscale approaches involving a Quantum Processing Unit (QPU) acceleration within the QM/QM scheme have been developed spanning several of the possibilities mentioned in Sec. 2.4. Particularly, refs (33 and 235−239) have demonstrated different approaches where the electronic structure of effective Hamiltonians is solved self-consistently updating on-the-fly the interaction terms on a classical computer. Depending on the different approach/implementation, the quantum computational budget may vary and, to date, a comprehensive numerical benchmark of these approaches is missing. Nonetheless, concerning the computational cost of including an external environment, it has already been shown that to account implicitly for the presence of an external environment does not increase the computational quantum budget.^229,231^ Also, regarding QM/MM strategies, the computational cost is defined by the size of the QM region and does not increase with respect to a gas phase calculation. Considering QM/QM schemes, again we do not expect differences as the environmental degrees of freedom can be adjusted on-the-fly while optimizing the variational circuit as it has already been done in QM/continuum approaches. We expect that the next years will be crucial for this area of research to estimate the effects of its practical impact. Future studies are necessary to bridge the gap between standard multiscale implementations and QPU-based strategies in order to address excited states description and a proper rationalization of the possible approaches to better understand which solution among the variational methods is more scalable. Lastly, it is important to specify that most of the mentioned studies benefit from software development kits that emulate quantum processors on classical architectures.^198,199,240−242^ To this end, we envisage that the continuous progresses in engineering highly efficient quantum emulators will be driven and simultaneously will drive new advances in multiscale modeling research for the coming decades.

##### 2.5.2.2 Quantum Multiscale Modeling on EFTQC

Although practically pertinent to the future of quantum processors, this area represents one of the current frontiers in quantum algorithms development.^215^ Unlike what we have seen above, EFTQC algorithms do not use the computer to efficiently sample (and navigate) a large computational space, but rather try to extract information by simulating the systems’ dynamics on the computer. The algorithms that fall into this section use circuits with few (if not just one) ancillary qubits.^243^ Due to the presence of two entangled qubit registers, methods that rely on this construction are defined as interference methods. In this regime, an attempt is made to develop methods that are flexible in terms of the ratio of quantum circuit depth to number of circuit repetitions.^244^ Such a tunable scaling spans different regimes going from a pure  number of circuit repetitions to the Heisenberg limit (optimal scaling for quantum information processes) of . Given the youth of this field, no multiscale methodologies have been developed yet, and empirical scalings of these methods for gas-phase systems remain unexplored. A recent example is the combined application of the algorithms of refs (245 and 246) to simulate the H_2_ molecule in gas phase.^247^ Notably, incorporating the nonlinear nature of multiscale problems as addressed in many of the mentioned approaches (see Sec. 2.1 to 2.4), will strongly depend on the structure of the algorithm: when a feedback loop between the CPU and the QPU is present for the isolated molecule the inclusion of an external environment could take place at no additional cost as the environment response is adjusted while the quantum algorithm is running.

##### 2.5.2.3 Quantum Multiscale Modeling on FTQC

To date, in the field of quantum computation on FTQC, the algorithm of choice, when applied to quantum simulation, is the Quantum Phase Estimation (QPE). In particular, much work focuses on the algorithmic cost analysis applied to chemically focused implementations.^248−250^ In this framework we aim to solve the eigenvalue estimation problem sampling on the quantum computer the autocorrelation function of the system and extracting its spectrum upon Fourier transform:
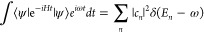
19

Here, *c*_*n*_ represents the overlap of the wave function |ψ⟩ with the eigenstate |*n*⟩, and *E*_*n*_ is the corresponding eigenvalue. For this reason its theoretical quantum advantage seems to be quite established as the overall quantum circuit is built combining (i) quantum simulation routines^251,252^ (for which theoretical quantum advantage is proved) and (ii) Quantum Fourier Transform (quadratic speedup compared the best-known classical Fast Fourier Transform algorithm) and (iii) different postprocessing of the results.^253,254^ Nonetheless, we remark that the effect of the initial state preparation^255−257^ may hamper the overall runtime efficiency^258^ and that the future application of this algorithm will strongly depend on hardware, architecture, and algorithm development.^259^

Being a very young field of research, the application of these methods to extended systems using multiscale techniques is not yet widely explored. It is however worth mentioning how in ref (232) a QM/MM scheme is applied also with a QPE algorithm. The PCM model has also been used to estimate the QPE computational cost for a protein–ligand binding system.^260^ In contrast to variational methods, incorporating this technique into standard strategies, which involve self-consistent loops, is not straightforward. The high computational cost of these circuits is challenging even for the medium-sized quantum computers that are expected to be developed in the coming decades. For this reason, our perspective on the development of these methodologies involves the inclusion of the degrees of freedom relevant to the external environment directly within the quantum Hamiltonian encoded into the QPU. Possible strategies include the works of refs (41, 261, and 262).

To conclude this section we want to remark that quantum computation is a technology which will be of practical use only if we will be able to answer affirmatively to several interdisciplinary questions, such as (i) what is the most efficient physical implementation of a quantum computer? (ii) How do we devise a scalable architecture? (iii) Considering it is an energy-intensive technology, can QC be energetically sustainable? (iv) Can we make quantum programming as popular as standard coding with user-friendly compilers and languages? (v) Can we actually develop efficient algorithms that outperform classical ones? All these questions and related answers will shape the future of quantum simulation and, in turn, of multiscale modeling.

## 3 Our Future Vision

Up to this point, we have analyzed several methods falling within the realm of multiscale modeling. As shown in the previous sections, the described landscape is extremely vast and diversified. Combining different levels of theory, from QM/Continuum to QM/QM, allows us to accurately assess phenomena that cannot be practically described with a full QM approach. As mentioned, over the last few decades the development of the discussed methods has increased our ability to study challenging systems and expanded our knowledge in many scientific fields.^22,29,42^ However, many phenomena still present insurmountable challenges for modern computational techniques. For instance, light-induced catalysts, such as those involved in natural and artificial photosynthesis, cannot be properly modeled to date. Natural photosynthesis encompasses all processes enabling plants and bacteria to convert sunlight energy into energy-rich biomolecules used to fuel other cellular processes.^263,264^ This phenomenon involves inherently multiscale reactions where, in most cases, enzymes act as electrostatic and confining envelopes around the reaction centers where quantum processes occur. These reactions begin with photon absorption and the translocation of the wavepacket to the reaction center. Artificial photosynthesis pursues the same goal, using transition metal materials as photoreceptors and photocatalysts instead of enzymes.^265^ Consequently, a profound and comprehensive understanding of the role of the environment around the reaction center becomes essential. This endeavor has been pursued over the last decades primarily using QM/MM.^29,42,264,266^ However, limitations of modern methods, whether on the simulation time scale or size, have restricted studies to compartmentalized examinations of single molecular events, such as charge,^266^ exciton,^29^ or proton transfer^267^ between only two or a few partners. The presence of robust computational tools (such as MLIPs, see Sec.2.5.1) that overcome such limitations would not only provide a more comprehensive understanding of natural processes but also serves as a powerful tool to study novel materials to achieve artificial photosynthesis. All these studies would lay the foundation for a green-based energetic revolution in the coming decades.^268^

**Figure 7:**
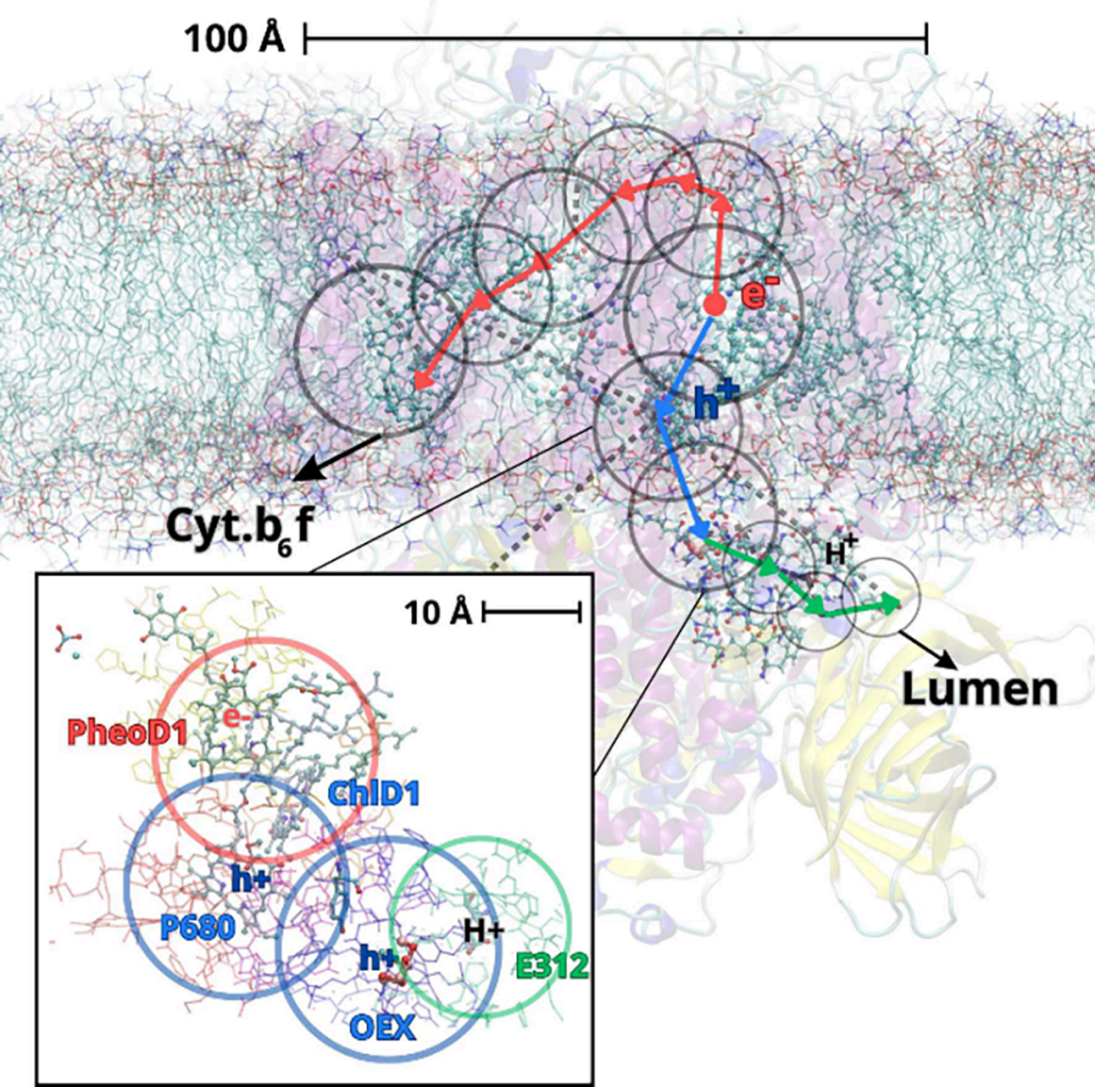
Schematic representation of the charge separation pathways in PSII. The displacement of protons (H^+^), electrons (e^–^), and holes (h^+^) is depicted. The initial charge separation occurs between Pheophytin-D1 (PheoD1) and Chlorophyll-D1 (ChlD1) couples. Subsequently, the electron moves from chromophore to chromophore, forming a reduced plastoquinone and being released outside of the protein (indicated by red arrows). Meanwhile, h^+^ moves toward the chlorophylls pair P680 and eventually accumulates in the oxide cluster OEX (indicated by blue arrows). The excess charge is later released as a proton using a specific channel passing through the E312 residue, which acts as a proton gate (green arrows). The relative position of the mentioned residue and the scale of the system are depicted in the focus panel. Protein depicted from crystal structure data collected in ref (283).

Over the years, Photosystem-II (PSII), a large membrane protein, has served as an optimal model to assess the predictive capabilities of many computational methods,^29,266,267,269−274^ directly comparing them with the huge amount of available experimental data.^264,266,267,275^ PSII functions as a positive/negative charge separation engine. The reaction scheme consists of funneling light-induced excited states into one part of the large reaction center, where the primary charge separation occurs. For each photon, one electron moves away from the reaction center (RC) jumping from chromophore to chromophore, initially providing a radical anion (A^–•^) and then a neutral-reduced cofactor, and eventually reaching the outside of the protein (before jumping to another one). On the other side, the electronic hole moves initially as a radical cation C^+•^ and then accumulates in a metal-oxide cluster named OEX (Oxygen Evolving Complex). The excess positive charge is balanced by the release of protons from water molecules attached to the cluster on the internal side of the membrane (Lumen), through dedicated proton channels. After every four photons, a catalytic cycle is completed, and an oxygen molecule obtained by the oxidation of two water molecules is released by the OEX. A simplified reaction scheme of the electron/hole movement is depicted in Figure 7. Therefore, the PSII reaction scheme involves purely quantum events such as charge/exciton transfer,^266,269,270,272,276,277^ semiclassical events like proton relocation,^267,274,278−280^ and classical events that do not involve bond breaking/formation, such as water supply and oxygen release. Additionally, all of these processes occur over tens to hundreds of Å, a scale that significantly challenges the actual multiscale implementations. Furthermore, some of these phenomena still extend beyond current computational power as they would require QM simulations to span in the ns time scale,^267,273,281,282^ while the MM part should account for heavy atom motion occurring in the μs or ms time scales. In this regard, some promising results have been recently achieved using MLIPs techniques, as discussed in Sec.2.5.1.

A domain large enough to envelope all the molecular moieties involved in QM events, includes up to tens of thousands of atoms. Such a large QM partition, together also with a continuum or atomistic environment, will probably not be available in the next decades and, perhaps, we do not even want it. First, due to the enormous computational cost of such simulations, which would also require a giant energetic expense;^284^ second, because it could also be considered a waste of computational resources. Indeed, several quantum properties have been shown to converge using only a few hundred QM atoms around the main actor of the reaction. Nowadays, a QM region that includes 200 to 300 atoms is usually considered large enough for most calculations, and anything beyond this threshold would be considered more than remarkable. Nevertheless, convergence evaluations for several properties have shown that, to achieve stable, reliable, and predictive results, a range from 300 to 500 atoms^285−287^ to almost 1000 atoms^287^ should be used, depending on the studied properties. These calculations, which are not yet ordinarily affordable (if not out of reach) for standard architectures, could benefit from the integration of TPU-based implementations of electronic structure methods or from the deployment of this task to quantum processor units (see Sec.2.5). Additionally, for flexible systems such as proteins, some studies have shown that the time-dependent motion of the environment significantly influences the properties of the reaction center.^270,286,288,289^ The variability of these motions cannot be adequately sampled within the time scales associated with QM/MM and so, multiple QM/MM simulations are necessary to obtain robust results. Moreover, in many cases the results coming from those calculations cannot be directly compared with experimental measurements, but provide only a qualitative description of the studied process. If we consider also the small size of the QM regions, inadequate sampling of environmental interactions, nonstandardized setups of multiscale simulations, and compartmentalization of studies focusing only on localized events, we obtain a rather discouraging view of the most significant problems with this methodology. These difficulties make the application of QM/MM on phenomena like photosynthesis really challenging, preventing an effective interpretation of the results and a direct comparison with the experimental data.

Several improvements have been recently proposed to tackle some of these challenges, starting with the establishment of a widely shared consensus on the setup procedure to initiate QM/MM simulations.^95,290,291^ A foolproof process would ensure enhanced reproducibility, subsequently increasing the predictive potential of calculations and enabling better management of approximations and associated errors. In the setup of multiscale simulations, an automated procedure for selecting an appropriate size for core QM region could play a crucial role, particularly in disordered systems like proteins, where radial selection may be intricate or even unnecessary. Achieving a rational and balanced approach between predictive capability and an atom-economical scheme remains the ultimate goal. Currently, the partitioning of the QM domain is mostly based on chemical insight, typically including mainly the first-shell residues from the protein environment. Other approaches have been proposed, involving the evaluation of a boundary based on the magnitude of the environment’s effect with Fukui shift analysis,^292^ electron density properties,^290,293^ and other strategies.^95,287,290^ In general, a systematic evaluation of the convergence of the quantum properties in the QM region should become best practice in setting up the calculations, while today it is still rare. A possible strategy to speed up this process without compromising the reliability of the partitioning scheme would be to use ML techniques that iteratively adjust the description of the different regions such as the ones discussed in Sec.2.5.1. The application of an adaptive QM region together with the possibility of moving the QM region (thereby changing the atoms treated at the QM level on-the-fly) would allow to study, in a single run, dynamical processes occurring over distances larger than a single QM region. A few pioneering studies have approached proton movement in bulk water,^294^ between acidic/basic residues in water,^295^ and also in a protein proton channel.^296^ For instance, the standard application of adaptive schemes to proton tranfer reactions applies an on-the-fly modification of the topology parameters when a residue leaving the previously defined QM region presents a different protonation state. The quantum region defined in this way is centered at the barycenter (*X*_*I*_: center of excess charge) between the hydrogen donor (*X*_*D*_) (i.e., heavy atoms where the hydrogen are located) and all the possible acceptors (*X*_*A*_):^295,296^
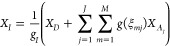
20where *m* and *j* are the possible donor/acceptor, *g*_*I*_ and *g*(ξ_*mj*_) are a weight functions and ξ_*mj*_ is the projection over the donor–acceptor vector.

In ion transfer studies, the minimal size of the QM region can be quite small, typically between 3 and 4 Å. In fact, the maximum displacement that a proton can make with a simple step, in a Grotthuss-like mechanism,^297^ is around 2 Å, while it can be considered a continuous displacement for heavier ions. The application of such a method would particularly thrive in electron/hole displacement and even more so for proton-coupled electron transfer (PCET), where the displacement of a proton is synchronized with an electron transfer. In PSII, the PCET occurring in the reaction center serves the positive/negative charge separation, establishing a chain of relocations for both protons and electrons up to the outside of the protein. The possibility of tracking a localized electron/hole using a similar approach has never been attempted, even though it could produce amazing results and additional insight in the nature of such processes.

Focusing on electron transfer (ET) events, they are commonly compatible with the actual simulation time scale. The main problem for ET is the partitioning of a proper QM center, as the features of electron donor/acceptor (D/A) moieties in a heterogeneous biomolecular system are not clearly defined. Moreover the intrinsic large size of both electron jumps and the D/A partners increase the computational demand of the simulation. The molecular species typically involved in ET can vary. Some biological examples include the Tyrosine side chain (9 atoms), isoalloxazine ring (∼30 atoms), plastoquinone ring (∼20 atoms), retinol (∼51 atoms), chlorophyll ring (∼80 atoms), pheophytin ring (∼80 atoms), and carotenoids (∼90 atoms). This is just to name a few, there are also many other artificial compounds that could be mentioned.^265^ Including at least three redox partners of such size within a single QM domain (to account for both backward and forward ET) would require treating thousands of atoms at the quantum level. Even considering a nonspherical region around the possible partners (an option that would require prior knowledge of the charge transfer path), this implementation is still beyond our computational capabilities. Nevertheless, the development of new architectures exploiting GPUs and TPUs could help scaling up the size of the problems under investigation, as described in Sec.2.5. This aspect represents also a possible goal for the development of scalable quantum simulation algorithms. The selection of the geometrical center of the QM region in ET processes is also challenging. In analogy with ion transfer,^296^ and considering the charge excess/defect as the variation from the expected value of charge density over each atom or group of atoms, the center of the QM domain could be considered, for instance, as the geometrical center of the atoms that differ the most with respect to a closed shell filling of the molecular orbitals. Similar to the center of excess charge, a possible *center of charge deviation* (CCD) could be considered as the geometrical average of the QM atoms’ positions, weighted by their deviation from their ground state condition. For example, if a tyrosine residue starts from a protonated state (R–OH, *g*_CCD_ = 0), the weight associated with the tyrosine atoms increases as the system approaches the density and orbital occupation corresponding to a full radical state (R–O^·^, *g*_CCD_ = 1). The velocity of the variation of such density (i.e., its time derivative) would also play a role. The size of the quantum region could be adjusted to a larger size for fast variation and to a smaller one for slow transition in order to accurately capture the evolution of the system’s properties. Such a methodological development would grant the possibility of investigating chemical events that still evade our computational ability, allowing an unbiased study of the time evolution of charge transfer processes over large spatial scales without relying on a first principle description of the entire system. The selection algorithm used to filter the right residues to be included in the quantum center could also benefit from ML methodologies. Leveraging the vast available databases would allow the real-time inclusion of the most important residues around the molecule acting as the center of the QM region. Additionally, when considering long-range interactions, the most relevant ones typically involve charged groups and their motion in space.^270,289,298,299^ Particularly, when studying processes composed of multiple events, the standard practice is to divide them, often neglecting the secondary effects of previous events or charge localization. Similarly, the PSII charge separation process, capable of moving charges over distances of almost 100 Å within the enzyme,^263,276^ has only been studied stepwise. The role of negative charge excess on one side of the charge transport versus the positive charge transport chain has never been assessed. Recent advancements in multicentric multiscale implementations provided the possibility of treating different regions of the total system at the QM level, both in QM/QM^300−302^ and in QM/MM frameworks.^303−305^ However, in this QM/MM approach, the QM region has to be far enough to be substantially independent, thus preventing any displacement or merging of such QM regions. The opportunity to use multiple QM regions, able to move and merge within a classical environment, will grant a new driving force for innovative studies of natural and artificial systems, boosting new technological applications. In this regard, over the past few years there has been a significant effort toward real-space and real-time tracking of localized excited state evolution and carrier dynamics in multichromophores aggregates. Such experimental and theoretical techniques make use of atomistically sharp plasmonic tips of scanning probe microscopes to manipulate and probe single molecules by combining tunneling electrons and visible light, thus achieving submolecular resolution thanks to plasmonic field effects.^306−311^ The presence of such metallic tips can be suitably modeled by Continuum methods (Figure 2b), hence we believe that a proper combination of the QM/MM novel implementations above-mentioned with a continuum description of plasmonic objects will pave the way for accurate space- and time-resolved analysis of energy funneling and transport dynamics over long paths in realistic light harvesting complexes.^29,312−400^ Once implemented, such ideas could open new horizons for computational studies in physical chemistry, that currently still rely on heavy and insight-based partitioning of the studied chemical problems, inevitably leading to discrepancies in the experimental-theoretical comparison. Furthermore, the modeling capabilities for quantum systems expected from the evolution of QC approaches are astonishing.^314,315^ The direct application of quantum-based calculators instead of classical ones promises a further leap toward higher accuracy and reliability in quantum calculations. The possibility of combining classical and quantum computers in order to harness the strengths of each (similar to CPU and GPU usage in current-era calculations), could introduce a novel concept of multiscale computation. This concept not only spans diverse levels of theory but also different algorithmic paradigms.

A seamless combination of various theoretical levels, from QC to CG models and continuum methods, could lead to the ultimate realization of the multiscale concept. The idea of dynamically switching in real-time between these levels, aided by the emergence of ML based methods, serves as the impetus for modeling extremely large systems (comprising over millions of atoms).^316,317^ This approach allows the core quantum region, not confined to a predetermined position, to benefit from the extensive computational model size, following the concept of studying organelles and cells in their entirety. While higher-level quantum regions encapsulated within lower ones are already feasible,^79^ and compatibility between MM and CG regions exists,^105^ a coherent combination of all the discussed methods is still in progress.

The computational challenge of such implementations and their simulation cost will be on an unprecedented scale. The upcoming exascale era, coupled with the extensive parallelization efforts in the development of computational programs, will empower access to new, hitherto unexplored fields in multiscale simulations. Expanding our view, the advent of novel, combined, and robust methodologies to investigate light-induced phenomena will extend beyond the realms of natural photosynthesis. Photocatalysis, in general, holds the potential to spearhead the green revolution of the coming decades.^268^ Sunlight, being a clean and renewable energy source, facilitates the development of sustainable production processes for nitrogen-rich molecules in agriculture.^318^ Furthermore, it enables the regeneration of waste biomass into chemical compounds that can forge a new path toward a green and atom-economy-based future.^319^

## 4 Conclusions

As we look forward to a new era in physical chemistry, multiscale modeling continues to evolve, presenting opportunities and challenges that demand a deeper understanding and collaboration across multiple scientific disciplines such as biology, engineering, and computer science. This work explored recent advancements, innovative applications, and ongoing research in multiscale modeling, highlighting its critical role in addressing fundamental questions and solving real world problems. The usefulness of multiscale approaches is intrinsically rooted in its ability to deal with systems’ complexity, and this makes it an indispensable tool when facing some of the most urgent social demands, such as energy supply, health and drug discovery, sustainability, and technological innovation. We provided overview of the different flavours of state-of-the-art multiscale approaches, with special emphasis on the systems or scientific questions they are best suited to. Moreover, we tried to propose some research lines for its further theoretical advancement in the next decades, with the aim of completely exploiting spatial and temporal flexibility (and thus the method’s adaptivity to the processes under study) in the partitioning of the system in different domains. In particular, we suggested proceeding with the search of quantitative criteria that would extend such on-the-fly adaptive approaches beyond the simulations of reaction pathways for which some preliminary knowledge is available and mandatory. In this regard, the possibility of including multiple movable and flexible QM centers surrounded by a classically or continuum described environment with whom the QM regions interact will surely pave the way for unprecedented insights on natural and artificial light-harvesting, thus potentially leading to major breakthroughs in energy funneling and control. In this work we have been mainly focused on the possible theoretical advancements that, given its current potentialities, are likely to make multiscale methods an even more profitable tool in the next decades. Besides, we are also convinced that its relevance will increase and be further appreciated for several other reasons that is worth mentioning here. Since the diverse treatment of the system’s domains comes with a reduction of computational costs, multiscale methods can be regarded as more sustainable and accessible approaches, compared to fully ab initio alternatives. In fact, they allow one to avoid wasting computational resources and thus reduce the consumption of energy. Moreover, we think multiscale modeling is also the most natural way to enhance scientific understanding of poorly explored phenomena within physical chemistry. We tend to forget this because we are often fascinated and impressed by the realism (and elegance) conferred by the existing first-principles frameworks to treat molecules and materials. However, it is clear that basing computations on multiscale models controlled by few parameters with immediate physical interpretation, has the potential to produce a solid grasp upon the important phenomenology, at the expense of less relevant details. As we look to the future, we expect that this will be the maxim that accompanies an increasing number of researchers on their path toward increasingly informative simulations of molecular systems embedded in complex environments.

We also believe that modern computational technologies such as artificial intelligence and virtual reality devices, will contribute to the spread of multiscale codes with user-friendly interfaces, which in the near future could provide immediate interactive access to input definition, system manipulation, and output visualization, aided by advanced graphics and perhaps also extended reality platforms. On the basis of this, we can imagine a more intuitive yet accurate framework representative of multiscale modeling that could be smoothly integrated into ordinary classes and training of the next generation of chemistry students, as a means to put special emphasis on the role of the environment in mediating chemical phenomena. This concept, which will be efficaciously taught by leveraging new pedagogical visual tools, will make multiscale modeling a cornerstone of computational and physical chemistry in the future.
